# Economic Analysis of Pandemic Influenza Vaccination Strategies in Singapore

**DOI:** 10.1371/journal.pone.0007108

**Published:** 2009-09-22

**Authors:** Vernon J. Lee, Mei Yin Tok, Vincent T. Chow, Kai Hong Phua, Eng Eong Ooi, Paul A. Tambyah, Mark I. Chen

**Affiliations:** 1 Center for Health Services Research, National University of Singapore, Singapore, Singapore; 2 Department of Epidemiology and Public Health, National University of Singapore, Singapore, Singapore; 3 Biodefence Center, Ministry of Defence, Singapore, Singapore; 4 Department of Microbiology, National University of Singapore, Singapore, Singapore; 5 Lee Kuan Yew School of Public Policy, Singapore, Singapore; 6 Defence Science Organization National Laboratories, Singapore, Singapore; 7 Duke-National University of Singapore Graduate Medical School, Singapore, Singapore; 8 Department of Medicine, National University of Singapore, Singapore, Singapore; 9 Communicable Disease Center, Tan Tock Seng Hospital, Singapore, Singapore; University of Sao Paulo, Brazil

## Abstract

**Background:**

All influenza pandemic plans advocate pandemic vaccination. However, few studies have evaluated the cost-effectiveness of different vaccination strategies. This paper compares the economic outcomes of vaccination compared with treatment with antiviral agents alone, in Singapore.

**Methodology:**

We analyzed the economic outcomes of pandemic vaccination (immediate vaccination and vaccine stockpiling) compared with treatment-only in Singapore using a decision-based model to perform cost-benefit and cost-effectiveness analyses. We also explored the annual insurance premium (willingness to pay) depending on the perceived risk of the next pandemic occurring.

**Principal Findings:**

The treatment-only strategy resulted in 690 deaths, 13,950 hospitalization days, and economic cost of USD$497 million. For immediate vaccination, at vaccine effectiveness of >55%, vaccination was cost-beneficial over treatment-only. Vaccine stockpiling is not cost-effective in most scenarios even with 100% vaccine effectiveness. The annual insurance premium was highest with immediate vaccination, and was lower with increased duration to the next pandemic. The premium was also higher with higher vaccine effectiveness, attack rates, and case-fatality rates. Stockpiling with case-fatality rates of 0.4–0.6% would be cost-beneficial if vaccine effectiveness was >80%; while at case-fatality of >5% stockpiling would be cost-beneficial even if vaccine effectiveness was 20%. High-risk sub-groups warrant higher premiums than low-risk sub-groups.

**Conclusions:**

The actual pandemic vaccine effectiveness and lead time is unknown. Vaccine strategy should be based on perception of severity. Immediate vaccination is most cost-effective, but requires vaccines to be available when required. Vaccine stockpiling as insurance against worst-case scenarios is also cost-effective. Research and development is therefore critical to develop and stockpile cheap, readily available effective vaccines.

## Introduction

The influenza A (H1N1-2009) pandemic has been declared a pandemic by the World Health Organization (WHO) which has led to the activation of pandemic plans worldwide. These include development of candidate pandemic vaccines and stockpiling on antiviral drugs. However, it is not possible to predict with certainty when and what strain will trigger the next influenza pandemic. The influenza virus's changing behavior, acquisition of adaptive mutations, expansion of host range, emerging transmissibility of different strains among humans, and potential for genetic re-assortment raise concerns [1] even in the early stages of an apparently mild pandemic.

In recent years, H5N1 vaccines have been touted as a possible pandemic vaccination strategy to protect against a potential H5N1 pandemic strain [2], [3]. Following successful clinical trials, such vaccines are currently available and some countries have begun stockpiling them. Many countries are also developing prototype vaccines against the H1N1-2009 strain. Although these vaccines provide a critical element of pandemic preparedness for policy makers, the cost-effectiveness of such a strategy is unknown. In addition, the pandemic may not be caused by current strains and the vaccine may not be totally effective. Policy makers will therefore have to consider the cost-effectiveness of deploying a vaccination strategy in anticipation of a pandemic; due to the substantial investment in research and development, and manufacturing of vaccines.

While several reports have compared the cost-effectiveness of vaccination [4], or treatment and/or prophylaxis with antiviral drugs [5], [6] during a pandemic, few studies have evaluated the cost-effectiveness of pandemic vaccination strategies. This paper provides a comparison on the economic outcomes of vaccination and stockpiles of vaccines against treatment with antiviral agents only, in Singapore. Singapore is a modern city-state with a well-connected global travel network such that influenza can readily spread to Singapore from anywhere in the world within a short period. Our findings provide a framework of optimal strategies and considerations for national policy makers to plan for a future pandemic.

## Methods

**Figure 1:**
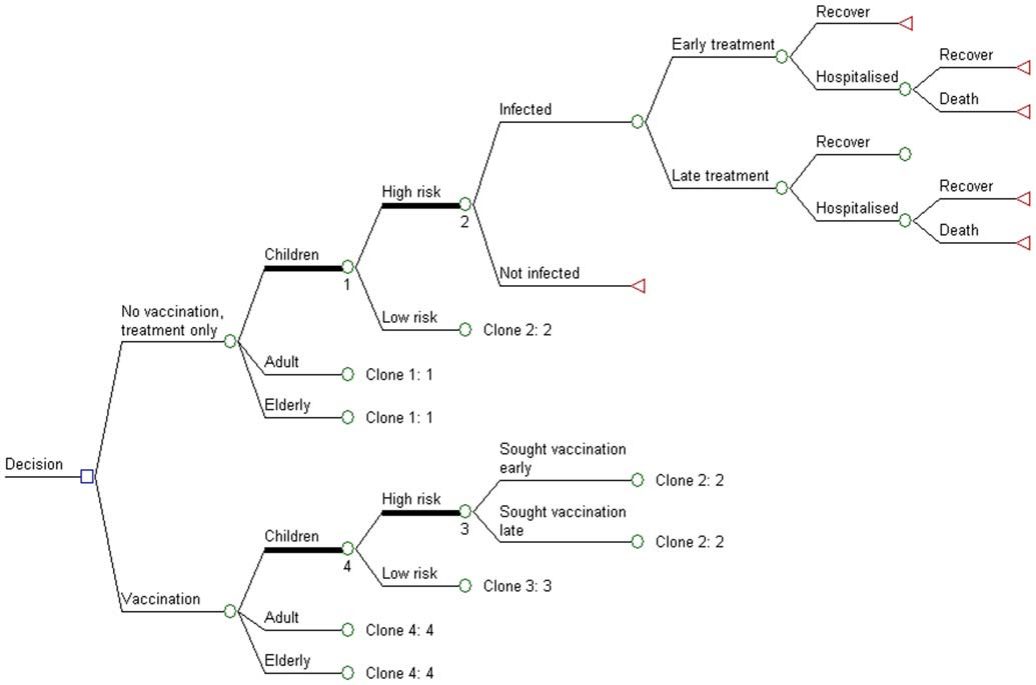
Decision diagram for vaccination versus treatment only.

Similar to a previous anti-viral study performed in Singapore [5], this study used a decision-analysis model (Figure 1) to perform cost-benefit and cost-effectiveness assessments for pandemic vaccination in Singapore. The model compared the current pandemic management strategy of early oseltamivir treatment and supportive management (treatment only) against pandemic vaccination in addition to early treatment (vaccination).

Cost-benefit analyses were performed to compare different vaccination strategies with treatment only, and included appropriate direct and indirect economic costs such as the cost of death. Cost-effectiveness analyses were also performed based on cost per-life-saved by vaccination and treatment only to provide another analytical dimension which avoids quantifying the value of life. The annual insurance premium was also used because it is difficult to predict a vaccine's true value. The annual insurance premium was the maximum cost that the country would be willing to pay annually to avert the impact of a pandemic, and includes all costs associated with vaccination including research and development, purchase, additional stockpiles, administration, and adverse effects. For example, a country would be willing to pay 3 times the annual insurance premium for a vaccine with 3 years shelf-life including administration, warehousing, and other costs. The analyses also provide the optimal strategies and investments for vaccines depending on vaccine efficacy and other key parameters.

### Input variables

The values for all parameters are shown in Table 1. The clinical variables are initially centered on a pandemic similar to the 1957 or 1968 pandemic, rather than the much more severe 1918 pandemic which will tend to favor additional intervention. The study was conducted based on Singapore's 2007 mid-year local population [7], divided into 3 age groups, each consisting of 2 risk groups [5].

**Table 1:** Input Variables Used in Analysis* †.

Input variables		Age Ranges		Sources
**Demographic data**	**0–19**	**20–64**	**≥65**	
Ave age	10	40	73	7
Population (thousand persons)	678.5	2,599	305.6	7
Low-risk	90%	89.7%	63.3%	
High-risk‡	10%	10.3%	36.7%	5
**Clinical data**				
Influenza clinical attack rate (%)	30 (10–50)	30 (10–50)	30 (10–50)	4,5,20
**Mortality**				
Case fatality (per 100,000)§				MOĤ, 5
Low-risk	5 (1–12.5)	6 (1–9)	340 (28–680)	
High-risk	137 (12.6–765)	149 (10–570)	1700 (276–3400)	
Earnings lost per death (USD$)¶	936,319	873,046	91,865	MOMˆ̂, [32]
**Hospitalizations**				
Hospitalization rate (per 100,000 infected)#				MOĤ
Low-risk	210 (42– 525)	72 (12–108)	1634 (135–3268)	
High-risk	210 (100 –1173)	234 (16–895)	2167 (352–4334)	
Average Length Of Stay (days)	3.88 (2.3 –9.2)	4.61 (3.2–11.8)	6.2 (4.6–13.4)	4,5,20
Average Additional Days Lost	2 (1–3)	2 (1–3)	2 (1–3)	Local physicians
Hospital cost (USD$/day)	284	284	284	MOĤ
Value of 1 lost day (USD$)*	55	84	55	MOMˆ̂, [32]
**Outpatient**				
Days lost from outpatient influenza	3 (1–5)	3 (1–5)	3 (1–5)	4,5,20
Consultation and treatment cost (USD$)	27	27	27	Local physicians
Value of 1 lost day(USD$)**	55	84	55	MOMˆ̂, [32]
**Treatment with oseltamivir**				
Sought early medical care (%)	70 (50–90)	70 (50–90)	70 (50–90)	5,20
Mortality reduction (%)	70 (50–90)	70 (50–90)	30 (20–90)	5,20
Hospital reduction (%)	60 (50–90)	60 (50–90)	30 (20–90)	5,20
Lost days gained (days)	1.0 (0.1–2.0)	1.0 (0.1–2.0)	1.0 (0.1–2.0)	5,20
**Vaccination**				
Sought early vaccination (%)	70 (50–90)	70 (50–90)	70 (50–90)	4,5
Cost of vaccination, (USD$)††	46.4	46.4	46.4	Estimated, 4, 10–12
% effective in preventing infection	60 (40–80)	60 (40–80)	0.48 (0.32–0.64)	10–18
Strain mismatch (%)	0 (0–100)	0 (0–100)	0 (0–100)	Estimated
**Economic Data**				
Mean Monthly Nominal Earnings 2007		USD$2,504		MOMˆ̂
Wage growth rate 2007		4.4%		MOMˆ̂
Discount rate		3%		

*Base case values are given with the range used for analysis given within the brackets ( ) where applicable. The input variables were modeled as triangular distributions centered on the base values with the minimum and maximum values given by the extreme values in the ranges.

†All healthcare costs were converted to 2007 US dollars and were compounded using the consumer price index for Singapore^6^.

‡High risk includes asthma, chronic obstructive pulmonary diseases, heart, and diabetes patients.

^Ministry of Health, Singapore.

^̂Ministry of Manpower, Singapore.

§Based on mortality among those having clinical influenza.

¶Average present value of future earnings lost per death of an individual of average age in the age group: using the 3% discount rate, of expected lifetime earnings and housekeeping services, weighted by age and adjusted to 2007 SGD$ dollars (multiplying by a factor of 0.24) [9].

#The rate is based on hospitalizations among those having clinical influenza. The ranges are calculated based on a factor of the base cases versus the mortality rate.

**USD$84 for lost work day, USD$55 for unspecified days lost (taking care of ill child/elderly, and additional days lost post-hospitalization).

††USD$20 per dose assuming 2 doses were required, and USD$7 for costs of administration and side effects.

Since several countries, including Singapore, have stockpiled oseltamivir as part of the preparedness for an influenza pandemic, we assumed that antiviral treatment is available for every resident, has been purchased *a priori* (i.e. sunk costs), and is a non-recurring cost. We then evaluated if the addition of pandemic vaccines to the treatment-only strategy provided a net cost benefit. Treatment is assumed to be given to all influenza-like-illness cases, regardless of vaccination. As oseltamivir treatment is optimal when administered early within 48 hours [8], [9], we assumed that only the proportion of patients who are treated in a timely manner would benefit from treatment.

### Pandemic Vaccine

As pandemic vaccination has hitherto not been deployed for the actual prevention of a pandemic, its efficacy is largely unknown. This study relied on current animal and human immunogenic studies for H5N1 candidate vaccines to determine the possible efficacies of a candidate pandemic vaccine [10]–[17]. Even though a recent clinical trial found cross-reactivity to diverse H5N1 strains [18], a future pandemic strain is unknown, and cross-reactivity will vary between and among influenza subtypes [19]. To allow for critical uncertainty in this key parameter, we have elected to perform separate analysis at different vaccine effectiveness levels.

Vaccine cost, efficacy, and cross-reactivity (as mentioned above) are 3 key variables. As a base-case scenario, we assumed the vaccination cost of USD$46.4, i.e. based on higher-end pricing of USD$19.9 per dose (assuming 2 doses were required compared to USD$6.6 for one dose of seasonal vaccine), and USD$6.6 for costs of administration and adverse effects similar to a previous study [4]. We assumed that the safety of pandemic vaccines is similar to seasonal influenza vaccination [10]–[12]. The other key variable of efficacy against similar strains was considered, with the base vaccine efficacy set at 60% (the elderly having lower efficacy) [4]. We assumed that vaccine efficacy was similar across reduction in illness, hospitalization, and death as there is a lack of data suggesting otherwise. We varied these parameters as a whole rather than providing different stochastic variations which may be unrealistic (e.g. low reduction in hospitalization but high reduction in case-fatality). The third key variable was the likelihood that the vaccine would not have cross-reactivity against the pandemic strain (strain mismatch). The overall effectiveness of the vaccine was measured as follows:
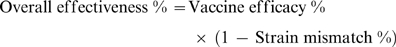



We also assumed that everyone within a population (or selected population sub-group) would be given the vaccine (i.e. stockpiling and delivery costs for the entire population or population sub-group), but that only the proportion that sought vaccination early enough for sufficient antibody development would benefit.

### Analysis

As pandemics are unpredictable with many uncertainties, we have modeled all uncertain input variables (Table 1) as triangular distributions centered on base values, with ranges based on the minimum and maximum plausible values derived from previous studies [4], [5]. Analyses were performed based on the key outcomes of overall cost-benefit, cost per life saved, and the annual insurance premium.

Multiple stockpiling scenarios were analyzed to observe the costs of different stockpile combinations. We explored scenarios where the pandemic arrives within the first stockpile (akin to vaccination for the current pandemic such as the H1N1-2009 without existing stockpiles), and after 10, 30, and 50 years, and we assumed that the stockpile would have a shelf-life of 3 and 5 years.

Sensitivity analyses were performed to identify variables that have the largest influence on the outcome. In addition, one-way sensitivity analyses were performed across several key variables such as the efficacy and cost of the vaccine. Monte Carlo simulation analyses were also performed to determine the key outcomes under 1,000 scenarios each. We also explored key outcomes by the 6 sub-groups to determine if pandemic vaccines would be particularly beneficial in any population sub-group if resources only allow for selected sub-groups to be vaccinated.

All costs were obtained and standardized to 2007 Singapore dollars, and represented in United States dollars using the following exchange rate (2007 exchange rate, USD$1: SGD$1.507). The model was run using Excel spreadsheets (Microsoft Corp, Redmond, WA) and @Risk (Palisade, Newfield, NY) simulation add-in. Details of the model and results are shown in the Technical Supplement.

## Results

If treatment-only strategy were adopted during a pandemic, the mean number of simulated deaths in Singapore is 690 (5^th^ and 95^th^ percentiles of 350 and 1,122), with 78% of deaths occurring in the high-risk group. The mean number of hospital days is 13,950 (7,360, 23,445) with a total of 2.5 million workdays equivalent lost (1.3 million, 4.1 million). The mean economic cost is USD$469.8 million (283.3 million, 1,303.9 million).

From the sensitivity analyses, the outcome was most sensitive to the case-fatality rate, followed by the attack rate (Figure S1). The key vaccine parameters of cost, efficacy, and strain mismatch also had a substantial impact on the outcome.

If the vaccine was to be used for an impending pandemic (within the first stockpile), the outcomes for a vaccine cost of USD$46.4, based on different levels of vaccine efficacy and cross-reactivity, are shown in Table 2. If the vaccine has a good match with the pandemic strain, the cost-benefit of vaccination compared to treatment only increased by USD$31.2 million (5^th^ and 95^th^ percentiles – USD$18.6 million and USD$50.4 million) while the number of deaths was reduced by 41 (21, 69) for every 10% increase in vaccine efficacy. At vaccine efficacies of >55%, vaccination was cost-beneficial over treatment-only. The mean cost per life saved decreased with increasing vaccine efficacy, and treatment-only was less beneficial than vaccination when the vaccine efficacy exceeded 85%. However, the effectiveness changes once strain mismatches (lack of cross-reactivity) are taken into account. If the strain mismatch was 20%, vaccination was cost-beneficial only at vaccine efficacies of >65%; if the mismatch was 40%, vaccination was cost-beneficial at efficacies of >82%; while vaccination was not cost-beneficial if the mismatch was >50%.

**Table 2:** Costs and outcomes for with changes in vaccine efficacy and strain mismatch (shown for vaccination within first stockpile)* †.

	**Cost benefit (millions USD$)**				
	**Strain mismatch**				
**Vaccine Efficacy**	**0.0**	**0.2**	**0.4**	**0.6**	**0.8**
0.2	103 (66, 128)	116 (87, 138)	126 (105, 142)	139 (124, 150)	151 (144, 157)
0.4	39 (−35, 90)	65 (9, 109)	86 (44, 118)	111 (81, 134)	135 (121, 147)
0.6	−24 (−136, 52)	14 (−70, 80)	46 (−17, 93)	83 (38, 118)	119 (98, 137)
0.8	−88 (−237, 14)	−37 (−149, 51)	5 (−78, 69)	56 (−4, 102)	104 (75, 127)
1.0	−152 (−338, −24)	−88 (−228, 22)	−35 (−139, 45)	28 (−47, 86)	88 (52, 117)
	**Lives saved**				
	**Strain mismatch**				
**Vaccine Efficacy**	**0.0**	**0.2**	**0.4**	**0.6**	**0.8**
0.2	82 (41, 138)	64 (32, 108)	50 (25, 85)	33 (17, 54)	16 (8, 26)
0.4	165 (82, 277)	129 (63,215 0	101 (51, 171)	65 (33, 109)	32 (17, 53)
0.6	247 (123, 415)	193 (95, 323)	151 (76,256)	98 (50, 163)	48 (25, 79)
0.8	330 (165, 553)	258 (127,431)	201 (102, 341)	130 (66, 218)	65 (33,105)
1.0	412 (206, 692)	322 (158, 539)	252 (127, 426)	163 (83, 272)	81 (42, 132)
	**Cost per life saved (millions, USD$)**				
	**Strain m6ismatch**				
**Vaccine Efficacy**	**0.0**	**0.2**	**0.4**	**0.6**	**0.8**
0.2	1.78 (0.86, 3.19)	2.42 (1.16, 4.37)	3.24 (1.62, 5.81)	5.23 (2.72, 9.05)	10.94 (5.85, 18.72)
0.4	0.63 (0.17, 1.23)	0.94 (0.37, 1.81)	1.34 (0.60, 2.45)	2.31 (1.15, 4.09)	5.10 (2.69, 8.80)
0.6	0.24 (−0.12, 0.64)	0.44 (0.04, 1.00)	0.71 (0.23, 1.41)	1.34 (0.60, 2.50)	3.15 (1.60, 5.52)
0.8	0.05 (−0.29, 0.36)	0.2 (−0.18, 0.61)	0.39 (0.01, 0.88)	0.85 (0.32, 1.71)	2.18 (1.08, 3.87)
1.0	−0.06 (−0.41, 0.21)	0.05 (−0.32, 0.39)	0.20 (−0.14, 0.58)	0.56 (0.12, 1.22)	1.59 (0.75, 2.85)

*Mean values are shown with 5^th^ and 95^th^ percentiles.

†All healthcare costs are in 2007 Singapore dollars.

If vaccines were stockpiled for a future pandemic, the outcomes of the analyses are shown in Appendix S1. In the scenario where the next pandemic occurred in 10 years and vaccine shelf-life was 5 years, it is evident that long term stockpiling of vaccines is not cost-effective in the mean scenario even with 100% vaccine efficacy and strain matching, and is only cost effective at the 5^th^ percentile with 100% vaccines efficacy and up to 20% strain mismatch.

Two-way sensitivity analyses were also conducted to determine the annual insurance premiums of the different strategies under different attack rates, case-fatality rates, and overall vaccine effectiveness; these are the key epidemic parameters which generate the greatest uncertainty in the outcome. Considering the maximum annual insurance premium based on vaccine stockpiling, the premium was higher with higher overall vaccine effectiveness and attack rates (Table 3). For vaccination within the first stockpile cycle, the assumed cost of vaccine of USD$46.4 is less than the maximum insurance premium when the attack rate is >38% at vaccine effectiveness of 40%; >25% at vaccine effectiveness of 60%; and >18% at vaccine effectiveness of 80%, suggesting that vaccination within the first stockpile cycle is cost-beneficial under these conditions. If vaccination costs are reduced to seasonal vaccination levels of about USD$6.6, vaccination within the first stockpile cycle is almost always cost-effective. Even under the lower cost of USD$6.6, long term stockpiling strategies were cost-beneficial only with high vaccine effectiveness of >60% or high attack rates of >40%.

**Table 3:** Annual insurance premium for pandemic scenarios with changes in vaccine effectiveness and attack rate* †.

	**Impending pandemic**			
	**Vaccine effectiveness**			
**Attack rate**	**0.2**	**0.4**	**0.6**	**0.8**
0.1	6.02 (4.13, 8.33)	11.8 (8.03, 16.2)	17.9 (12.5, 24.3)	23.8 (16.4, 32.6)
0.3	18.1 (12.4, 25.0)	35.5 (24.1, 48.5)	53.8 (37.5, 72.7)	71.4 (49.3, 97.9)
0.5	30.1 (20.7, 41.7)	59.2 (40.2, 80.9)	89.6 (62.5, 121.2)	118.9 (82.2, 163.2)
	**Probability of pandemic occurring spread over 10 years**			
	**Vaccine effectiveness**			
**Attack rate**	**0.2**	**0.4**	**0.6**	**0.8**
0.1	0.60 (0.41, 0.83)	1.18 (0.80, 1.62)	1.79 (1.25, 2.42)	2.38 (1.64, 3.26)
0.3	1.81 (1.24, 2.50)	3.55 (2.41, 4.85)	5.37 (3.75, 7.27)	7.14 (4.93, 9.79)
0.5	3.01 (2.07, 4.16)	5.92 (4.02, 8.09)	8.96 (6.25, 12.1)	11.9 (8.22, 16.3)
	**Probability of pandemic occurring spread over 30 years**			
	**Vaccine effectiveness**			
**Attack rate**	**0.2**	**0.4**	**0.6**	**0.8**
0.1	0.20 (0.14, 0.28)	0.39 (0.27, 0.54)	0.60 (0.42, 0.81)	0.79 (0.55, 1.09)
0.3	0.60 (0.41, 0.83)	1.18 (0.80, 1.62)	1.79 (1.25, 2.42)	2.38 (1.64, 3.26)
0.5	1.00 (0.69, 1.39)	1.97 (1.34, 2.70)	2.99 (2.08, 4.04)	3.96 (2.74, 5.44)
	**Probability of pandemic occurring spread over 50 years**			
	**Vaccine effectiveness**			
**Attack rate**	**0.2**	**0.4**	**0.6**	**0.8**
0.1	0.123 (0.08, 0.17)	0.24 (0.16, 0.32)	0.36 (0.25, 0.48)	0.48 (0.33, 0.65)
0.3	0.36 (0.25, 0.50)	0.71 (0.48, 0.97)	1.07 (0.75, 1.45)	1.43 (0.99, 1.95)
0.5	0.60 (0.41, 0.83)	1.18 (0.80, 1.62)	1.79 (1.25, 2.42)	2.38 (1.64, 3.26)

*Mean values are shown with 5^th^ and 95^th^ percentiles.

†All healthcare costs are in 2007 US dollars (2007 exchange rate, USD$1: SGD$1.507).

**Figure 2:**
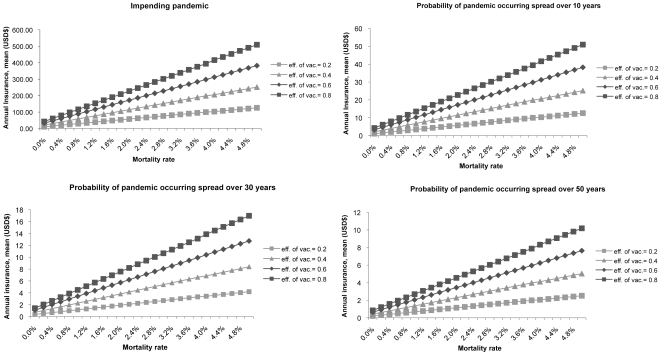
Annual insurance premium for pandemic scenarios with changes in vaccine effectiveness and mortality rate*†. *Mean values are shown with 5^th^ and 95^th^ percentiles. †All healthcare costs are in 2007 US dollars.

At case-fatality rates of 0.1% [similar to inter-pandemic epidemics [20]–[22]], most of the cost-benefit decisions favor treatment-only over long-term stockpiling (Figure 2). With the current influenza A (H1N1-2009) pandemic mortality of between 0.1 to 0.2% [23], [24], immediate purchase and vaccination will incur an insurance premium of between USD$25.9 to 30.6 for a vaccine with 40% effectiveness, USD$39.5 to 46.5 with 60% effectiveness, and USD$51.7 to 61.0 for a vaccine with 80% effectiveness. At the USD$46.4 estimated cost, a vaccine with 80% effectiveness would therefore be cost-effective.

For long term stockpiling strategies with 1957 and 1968 pandemic case-fatality rates of 0.4 to 0.6%, stockpiling would be cost-beneficial only if the pandemic occurred within 10 years, the shelf-life was 5 years, and vaccine effectiveness was >80%. As the case-fatality increases, the maximal annual insurance premiums match the USD$46.4 estimated costs at a wider range of permutations. At a case-fatality of 5% [the higher end of the 1918 pandemic [25]], stockpiling would be cost-beneficial even at probabilities of 50 years to the next pandemic if vaccine costs could be reduced to seasonal levels.

The population sub-group analysis considered the annual insurance premium when only a particular sub-group was vaccinated (Table 4). Protection of the high-risk sub-groups commanded a higher insurance premium compared to the low-risk sub-groups. For the high-risk sub-groups, children commanded the highest premiums followed by adults and the elderly. However, targeting the elderly resulted in the lowest cost per life saved, as well as the most lives saved by vaccination compared to treatment-only (Appendix S1).

**Table 4:** Mean annual insurance premium for pandemic scenarios with changes in vaccine effectiveness, by age and risk groups* †.

**Impending pandemic**				
**Vaccine effectiveness**	**0.2**	**0.4**	**0.6**	**0.8**
Low risk - children	9.43	18.9	28.3	37.7
Low risk - adult	12.8	25.6	38.4	51.2
Low risk - elderly	14.8	29.6	44.4	59.2
High risk - children	67.4	134.7	202.1	269.5
High risk - adult	57.3	114.6	172	229.3
High risk - elderly	45.7	90.9	136.4	181.8
**Probability of pandemic occurring spread over 10 years**				
**Vaccine effectiveness**	**0.2**	**0.4**	**0.6**	**0.8**
Low risk - children	0.94	1.89	2.83	3.77
Low risk - adult	1.28	2.56	3.84	5.12
Low risk - elderly	1.48	2.96	4.44	5.92
High risk - children	6.74	13.47	20.21	26.95
High risk - adult	5.73	11.46	17.19	22.93
High risk - elderly	4.55	9.09	13.64	18.18
**Probability of pandemic occurring spread over 30 years**				
**Vaccine effectiveness**	**0.2**	**0.4**	**0.6**	**0.8**
Low risk - children	0.31	0.63	0.94	1.26
Low risk - adult	0.43	0.85	1.28	1.71
Low risk - elderly	0.49	0.99	1.48	1.97
High risk - children	2.25	4.49	6.74	8.98
High risk - adult	1.91	3.82	5.73	7.64
High risk - elderly	1.52	3.03	4.55	6.06
**Probability of pandemic occurring spread over 50 years**				
**Vaccine effectiveness**	**0.2**	**0.4**	**0.6**	**0.8**
Low risk - children	0.19	0.38	0.57	0.75
Low risk - adult	0.26	0.51	0.77	1.02
Low risk - elderly	0.30	0.59	0.89	1.18
High risk - children	1.35	2.69	4.04	5.39
High risk - adult	1.15	2.29	3.44	4.59
High risk - elderly	0.91	1.82	2.73	3.64

*Mean values are shown with 5^th^ and 95^th^ percentiles.

†All healthcare costs are in 2007 US dollars (2007 exchange rate, USD$1: SGD$1.507).

## Discussion

We have shown that pandemic vaccination is only cost effective in severe pandemics, high vaccine effectiveness, and low vaccine cost. The outcome of the model was very sensitive to the overall vaccine effectiveness (a combination of vaccine efficacy and strain matching); and the vaccine cost. Vaccine manufacturers and policy-makers should be aware of the importance of these key vaccine development parameters and the trade-off between effectiveness and price, while noting that the economic outcomes are different between countries and should be based on local data. Vaccine efficacy may vary widely as shown by the efficacy of existing H5N1 candidate vaccines against different H5N1 strains [10]–[18]. The actual pandemic subtype could be different from existing candidate vaccines, increasing the unpredictability. More research is therefore required to develop candidate vaccines that provide wider cross-reactivity and cross-protection, or to develop vaccine libraries with multiple sub-types that can be quickly produced when a novel influenza virus is detected.

The differences in the outcomes where vaccination is deployed in the first stockpile cycle (immediate vaccination) versus some time during subsequent stockpile cycles will also have to be considered. As evident from Table 3, in the case of long term stockpiling wastage of earlier stockpiles occurs given the short shelf-life of the vaccines, with the result being generally less cost-beneficial, unless costs are reduced to seasonal vaccine levels or the perception of severity is high. It may seem more cost-beneficial when vaccines can be used within the first stockpile (purchasing vaccines for vaccination only when a pandemic is impending) which will also ensure the best strain match. The alternative strategy, which avoids the costs and problems associated with long-term stockpiling, is to await the appearance of a candidate pandemic strain before accumulating a vaccine stock, with a view to immediate vaccination once the vaccine stock becomes available. This is reflected in the influenza A (H1N1-2009) pandemic where there are no prior stockpiles and development of vaccines are underway. A vaccine for immediate vaccination against influenza A (H1N1-2009) with >80% effectiveness would be cost-effective at case-fatality rates as low as 0.1–0.2%. However, this strategy may be affected by the time required for vaccine development and availability before the pandemic infects a substantial proportion of the population, given the high global demand [26]. Safety of such a new vaccine would also have to be considered [27]. It may therefore be useful to develop low-cost vaccines that provide good cross-protection and lasting-immunity against a variety of influenza strains for long term stockpiling, of perhaps consider vaccines with good cross-protection for seasonal influenza vaccination programs.

Policy makers should consider funding research, development and local production of vaccines using annual insurance premiums as a guide to their willingness to pay for vaccine research and purchases to prevent a potential catastrophe. If vaccine stockpiling is to be considered, the cost of the vaccine is important. Other factors which must be considered are the cross-reactivity of the vaccine, context-specific political considerations (e.g. the value placed on efficient management of crisis events), the potential benefit of reducing surge demand for healthcare services through vaccination, and the effect of rapid developments in vaccine technology which may favor stockpiling, since with each stockpile cycle progress in vaccine technology may make future vaccines cheaper or more efficacious or both. For example, for an impending pandemic occurring within the first stockpile cycle (influenza A (H1N1-2009) pandemic), USD$61.0 may be spent per capita to obtain a vaccine that has an overall effectiveness of 80% against a pandemic with 0.2% case-fatality. For Singapore, this amount of >USD$199.1 million may be used for novel virus vaccine development or purchase agreements. However, for future pandemics, the premium decreases to USD$10.0 and USD$3.3 if the pandemic is estimated to occur over the next 10 and 30 years respectively. Development of cheaper and more effective vaccines is therefore necessary for long-term stockpiling to be feasible.

At high attack and case-fatality rates with low vaccine efficacy, the annual insurance premiums are much higher when compared to high vaccine efficacy but with low attack and case-fatality rates. If the perceived risk of disease severity is high (e.g. similar to the 1918 pandemic), the willingness to pay will be greater and even long term stockpiling will be cost-beneficial. The decision to purchase pandemic vaccines is therefore highly dependent on the perception of disease severity.

It is also evident that high-risk subpopulations would benefit most from vaccination due to the high number of lives saved. High-risk children have high case-fatality rates and the highest economic value from future earnings, and hence command the highest annual insurance premiums. Similarly, high-risk elderly showed the lowest cost per life saved due to the largest reduction in deaths. Even if nation-wide vaccination or stockpiling are not viable options; or if vaccines are insufficient for the entire population, strategies which prioritize vaccination of high-risk groups could be considered independently, with children and adults favored for the highest economic effectiveness and elderly favored for more overall lives saved. Finally, our analysis also showed that the proportion who received early vaccination was also important, and underscores the point that stockpiles must be accompanied by well-planned programs to rapidly administer vaccinations. The current Influenza A H1N1 2009 pandemic has shown that additional high-risk sub-populations may be identified. These have included pregnant women and obese individuals and additional studies are required to determine the input and outcome parameters for these sub-populations [28], [29].

Some limitations of this study include the exclusion of the societal value of health – although cost-utility analyses could address this, there are no available local indicators for Singapore. In addition, we have not included the pandemic's macro-economic impact which would likely favor interventions to a greater degree. While we have not considered recurring treatment stockpiles, any additional treatment stockpile costs will be borne equally by both strategies. For comparability, neither treatment nor vaccination was assumed to alter the pandemic's transmission dynamics. Vaccination, in particular, may reduce total population attack rates through increasing herd-immunity, but it is difficult to predict the impact of an imperfect pandemic vaccine on the transmission dynamics of a pandemic with characteristics that are still not fully defined [30], [31]. However, such immunity makes the argument for a pandemic vaccine even more compelling. We also did not consider long-term protection with immediate vaccination as that is associated with uncertainties including technological feasibility, waning immunity, and population turnover. Finally, while these findings are focused on Singapore, we believe that it provides a framework for other countries to consider analyzing in their own setting, which is crucial to determining local economic effectiveness.

Our study has shown that long-term vaccine stockpiling is expensive and may not be cost-effective at the population level for mild pandemics. However, stockpiling is cost-beneficial if insurance against a severe pandemic is the main priority, if specific high-risk groups are targeted, and if cheap and effective vaccines are rapidly available through research and development of novel vaccine technologies.

## Supporting Information

Appendix S1 Technical appendix with supporting information
(0.47 MB DOC)

Figure S1 (0.43 MB TIF)

## References

[1] Chow VT, Tambyah PA, Goh KT (2008). To kill a mocking bird flu?. Ann Acad Med Singapore.

[2] Carter NJ, Plosker GL (2008). Prepandemic Influenza Vaccine H5N1 (Split Virion, Inactivated, Adjuvanted) [Prepandrix(trade mark)]: A Review of its Use as an Active Immunization Against Influenza A Subtype H5N1 Virus.. BioDrugs.

[3] El Sahly HM, Keitel WA (2008). Pandemic H5N1 influenza vaccine development: an update.. Expert Rev Vaccines.

[4] Meltzer MI, Cox NJ, Fukuda K (1999). The Economic Impact of Pandemic Influenza in the United States: Priorities for Intervention.. Emerg Infect Dis.

[5] Lee VJ, Phua KH, Chen MI, Chow A, Ma S (2006). Economics of neuraminidase inhibitor stockpiling for pandemic influenza, Singapore.. Emerg Infect Dis.

[6] Balicer RD, Huerta M, Davidovitch N, Grotto I (2005). Cost-benefit of stockpiling drugs for influenza pandemic.. Emerg Infect Dis.

[7] Key statistics. Singapore Department of Statistics. Available from http://www.singstat.gov.sg/ Accessed 18 Sept 2008

[8] Moscona A (2005). Neuraminidase inhibitors for influenza.. N Engl J Med.

[9] Aoki FY, Macleod MD, Paggiaro P, Carewicz O, El Sawy A (2003). IMPACT Study Group. Early administration of oral oseltamivir increases the benefits of influenza treatment.. J Antimicrob Chemother.

[10] Levie K, Leroux-Roels I, Hoppenbrouwers K, Dramé M, Hanon E (2008). An adjuvanted, low-dose, pandemic influenza A (H5N1) vaccine candidate is safe, immunogenic, and induces cross-reactive immune responses in healthy adults.. J Infect Dis.

[11] Lin J, Zhang J, Dong X, Fang H, Chen J (2006). Safety and immunogenicity of an inactivated adjuvanted whole-virion influenza A (H5N1) vaccine: a phase I randomised controlled trial.. Lancet.

[12] Bresson JL, Perrone C, Launay O, Gerdil C, Saville M (2006). Safety and immunogenicity of an inactivated split virion influenza A/Vietnam/1194/2004 (H5N1) vaccine: phase 1 randomised trial.. Lancet.

[13] Leroux-Roels I, Bernhard R, Gérard P, Dramé M, Hanon E (2008). Broad Clade 2 cross-reactive immunity induced by an adjuvanted clade 1 rH5N1 pandemic influenza vaccine.. PLoS ONE.

[14] Baras B, Stittelaar KJ, Simon JH, Thoolen RJ, Mossman SP (2008). Cross-protection against lethal H5N1 challenge in ferrets with an adjuvanted pandemic influenza vaccine.. PLoS ONE.

[15] Leroux-Roels I, Borkowski A, Vanwolleghem T, Drame M, Clement F (2007). Antigen sparing and cross-reactive immunity with an adjuvanted rH5N1 prototype pandemic influenza vaccine: a randomised controlled trial.. Lancet.

[16] Treanor JJ, Campbell JD, Zangwill KM, Rowe T, Wolff M (2006). Safety and immunogenicity of an inactivated subvirion influenza A (H5N1) vaccine.. NEJM.

[17] Stephenson I, Bugarini R, Nicholson KG, Podda A, Wood JM (2005). Cross-reactivity to highly pathogenic avian influenza H5N1 viruses after vaccination with nonadjuvanted and MF59-adjuvanted influenza A/Duck/Singapore/97 (H5N3) vaccine: a potential priming strategy.. J Infect Dis.

[18] Ehrlich HJ, Müller M, Oh HM, Tambyah PA, Joukhadar C (2008). A clinical trial of a whole-virus H5N1 vaccine derived from cell culture.. N Engl J Med.

[19] Influenza Team European Centre for Disease Prevention and Control (ECDC) (2007). Human influenza A/H5N1 (“pandemic”) vaccines: informing policy development in Europe.. Euro Surveill.

[20] Turner D, Wailoo A, Nicholson K, Cooper N, Sutton A (2003). Systematic review and economic decision modeling for the prevention and treatment of influenza A and B.. Health Technol Assess.

[21] Ng TP, Pwee TH, Niti M, Goh LG (2002). Influenza in Singapore: assessing the burden of illness in the community.. Ann Acad Med Singapore.

[22] Viboud C, Boelle PY, Pakdaman K, Carrat F, Valleron AJ (2004). Influenza epidemics in the United States, France, and Australia, 1972–1997.. Emerg Infect Dis.

[23] Centers for Disease Control and Prevention (CDC) (2009). Human infection with new influenza A (H1N1) virus: clinical observations from Mexico and other affected countries, May 2009.. Wkly Epidemiol Rec.

[24] Lipsitch M, Riley S, Cauchemez S, Ghani AC, Ferguson NM (2009). Managing and Reducing Uncertainty in an Emerging Influenza Pandemic.. N Engl J Med.

[25] Glezen WP (1996). Emerging infections: pandemic influenza.. Epidemiol Rev.

[26] Collin N, de Radiguès X (2009). World Health Organization H1N1 Vaccine Task Force. Vaccine production capacity for seasonal and pandemic (H1N1) 2009 influenza.. Vaccine.

[27] Evans D, Cauchemez S, Hayden FG (2009). “Prepandemic” immunization for novel influenza viruses, “swine flu” vaccine, guillain-barré syndrome, and the detection of rare severe adverse events.. J Infect Dis.

[28] Jamieson DJ, Honein MA, Rasmussen SA, Williams JL, Swerdlow DL (2009). H1N1 2009 influenza virus infection during pregnancy in the USA.. Lancet. In Press.

[29] Centers for Disease Control and Prevention (CDC) (2009). Intensive-care patients with severe novel influenza A (H1N1) virus infection - Michigan, June 2009.. MMWR Morb Mortal Wkly Rep.

[30] Riley S, Wu JT, Leung GM (2007). Optimizing the dose of pandemic influenza vaccines to reduce the infection attack rate.. PLoS Med.

[31] Palache B (2008). New vaccine approaches for seasonal and pandemic influenza.. Vaccine.

[32] Haddix AC, Teutsch SM, Coros PS (2002). A guide to decision analysis and economic evaluation. 2nd Edition..

