# Supplementary material for: Economic Analysis of Pandemic Influenza Vaccination Strategies in Singapore
Source: PLoS One. 2009 Sep 22;4(9):e7108. doi: 10.1371/journal.pone.0007108 (PMC2743808; doi:10.1371/journal.pone.0007108)
Supplement: Appendix S1 — Technical appendix with supporting information (0.47 MB DOC) [file pone.0007108.s001.doc]

**Appendix S1**

**Economic Analysis of Pre-pandemic Influenza Vaccination Strategies in Singapore**

Vernon J Lee, Mei Yin Tok, Vincent T Chow, Kai Hong Phua, Eng Eong Ooi, Paul A Tambyah, Mark I Chen

**Introduction**

Based on the interest by countries to purchase, stockpile, or deploy prototype pandemic vaccines in anticipation of a pandemic. To address this issue from a healthcare and economics angle, we developed an economic evaluation model to determine the cost-effectiveness of a vaccination strategy. This technical appendix serves to provide a detailed rationale for the methods and additional results and findings that provide additional support to the conclusions.

**Methods**

The decision-analysis model used in this study was similar to our previously published work on anti-viral stockpiling strategies in Singapore (1). The current model compared the de facto pandemic management strategy of early oseltamivir treatment and supportive management (treatment only) against pandemic vaccination in addition to early treatment (vaccination). Treatment with anti-virals have been shown to be a cost-effective strategy across a wide range of scenarios (1,2). Different scenarios of pandemic vaccination were considered - vaccination within the first stockpile cycle (akin to immediate vaccination for an impending pandemic), or long-term stockpiling of candidate vaccines which will be deployed when a pandemic is imminent. The scenario of vaccination within the first stockpile cycle was included due to the consideration for immediate vaccination with a prototype vaccine against an impending pandemic, for example the 1976 Influenza A H1N1 “Swine Flu” or the 2009 Influenza A (H1N1), or a vaccine which can provide sufficient cross-reactivity against likely impending pandemic sub-types. The long term stockpiling scenario is included because several countries had started or were planning on stockpiling H5N1 candidate vaccines, and this could be applicable to other sub-types which have pandemic potential. For the long term stockpiling scenario, we included the time to the next pandemic of 10, 30, and 50 years as these are within the frequency between pandemics observed across history. In addition, we assumed that the stockpile would have a shelf-life of 3 and 5 years depending on the manufacturing and storage process (for example storing the active ingredient and the adjuvant separately).

Pandemic severity

We used base parameters which were more similar to the 1957 or 1968 pandemic, rather than the much more severe 1918 pandemic. This is because higher case-fatality rates will favor costly interventions such as anti-viral prophylaxis or vaccination. However, much of the preparations against the next pandemics is for high case-fatality and we have included sensitivity analyses to explore the cost-effectiveness of these strategies at various case-fatality rates.

Pandemic Vaccine

The efficacy of candidate pandemic vaccines during an actual pandemic is unknown. We relied on existing studies to determine the effectiveness of influenza vaccines against matched strains. However, a candidate pandemic vaccine may not have a perfect match with the pandemic strain due to mutations and reassortments resulting in antigenic drifts or shifts. We therefore considered the vaccine efficacy independently from a match between the candidate pandemic vaccine where cross-reactivity is assumed to occur, versus a mismatch between strains in which there is no cross-reactivity and the vaccine does not provide any protective effect.

The vaccine cost, efficacy, and cross-reactivity (strain matching) are 3 key variables. The vaccine cost is based on the estimated costs for vaccination including administration and side effects. The vaccine cost is used as a baseline to compare the different strategies and in the sensitivity analyses. However, we included an addition analysis – the annual insurance premium, which describes the maximal cost or willingness to pay for the vaccine based on the impact caused by the pandemic.

Efficacy against similar matched influenza strains was also considered, and since this is one of the key variables of contention for an untested vaccine, we performed numerous sensitivity analyses on a wide range of values from 0 to 100% to provide an complete representation of the impact of this variable since new vaccines may not be fully efficacious against a similar matched strain. We assumed that vaccine efficacy was similar in the reduction in illness, hospitalization, and death in the absence of data suggesting otherwise. We varied these parameters as a whole rather than providing different stochastic variations which may be unrealistic (e.g. low reduction in hospitalization but high reduction in case-fatality).

The third key variable was the likelihood that the vaccine would have cross-reactivity against the pandemic strain (good strain matching). The actual pandemic strain may be very different from the pandemic strain and if such mismatch occurs, would not provide protection against the strain, regardless of the efficacy of the vaccine. This is possible because of the many influenza subtypes and strains, and the limitation of the current vaccine technology which focuses on the HA and NA surface antigens which readily mutate.

The overall effectiveness of the vaccine was the combination of efficacy and likelihood of strain matching (cross reactivity). We used this overall effectiveness as an aggregate for most of the annual insurance premium analyses because this is the important factor in vaccine development. Newer vaccines which target conserved regions of the influenza virus to provide good cross protection, and newer adjuvants which provide greater seroconversion and protection are part of the research and development that policy makers should be considering in the overall insurance costs to prepare for the next pandemic. A vaccine with poor efficacy or poor cross reactivity will fare badly in the economic evaluation.

Analysis

Analyses were performed based on the key outcomes of overall cost-benefit, cost per life saved, and the annual insurance premium. The overall cost-benefit was used because it provided a common metric of comparison in Singapore dollars which provides an easy reference. Cost per life saved was used to provide an additional dimension due to the large number of lives lost and the relatively higher proportion of deaths in the elderly population. This elderly population has less direct economic value but is nevertheless an important part of society and has substantial intangible value.

The annual insurance premium was used because it is difficult to predict the vaccine’s true value. Similar to another study on the economic evaluation of pandemic preparedness strategies, we used the annual insurance premium to represent the maximum cost that the country would be willing to pay to avert the impact of the pandemic, and includes all costs associated with vaccination including research and development, purchase, additional stockpiles, administration, and adverse effects (3). The annual insurance premium is calculated as follows:

Annual insurance premium = net benefit from the strategy x the annual probability of a pandemic occurring

The net benefit from the strategy is calculated using the formulae described later for each of the key input variables. The net benefit is an aggregate of the costs incurred during a pandemic, and the benefits accrued as a result of the intervention strategy. The annual probability of the pandemic occurring is unknown and we assumed that a pandemic may be impending (immediate), or occur over the next 10, 30 or 50 years, corresponding to an annual probability of 1/10, 1/30, and 1/50 respectively.

Equations

All calculations done in the model had taken into consideration the effect of receiving vaccination early and the effect of timely oseltamivir treatment versus those who were vaccinated and treated late. The cost from influenza mortality, net present value of future earnings, cost of hospitalizations and cost of outpatient lost days are calculated as follows:

Cost from influenza mortality

*Economic cost from influenza mortality = Population _age, risk group_ x Attack rate x Case fatality _age, risk group_ x Net Present Value of Future Earnings _age, risk group_*

Cost of hospitalizations

The hospitalization cost for influenza-related complications was calculated by the summation of direct hospitalization cost with cost of additional days lost post-hospitalization. The direct hospitalization cost was calculated as follows:

*Economic cost of hospitalization _age, risk group_ = (Population _age, risk group_ x Attack rate x Hospitalization rate _age, risk group)_ x Length of stay _age, risk group_ x (Hospitalization cost + Value of a day lost _age, risk group_)*

The cost from additional days lost was calculated as follows:

*Economic cost of additional days lost post-hospitalization = Population _age, risk group_ x Attack rate x Hospitalization rate _age, risk group_ x Additional days lost _age, risk group_ x Value of a day lost _age, risk group_*

Cost of outpatient lost days

The cost of outpatient lost days was calculated using work days lost for the adult population and unspecified days lost for the young and elderly populations as follows:

*Economic cost of outpatient lost days _age, risk group_ = Population _age, risk group_ x Attack rate x Outpatient days lost x Value of a day lost _age, risk group_*

The overall net returns due to vaccinations, savings from outcomes averted and the costs of vaccination are calculated as follows:

Cost benefit compared to treatment only

*Cost-benefit = Overall cost _vaccination_ – Overall cost _treatment only_*

Cost effectiveness compared to treatment only

*Cost per-life-saved = (Cost excluding cost per life _vaccination_ – Cost excluding cost per life _treatment only_) / (Deaths _treatment only_ – Deaths _vaccination_)*

To determine the breakeven cost of vaccination under different vaccination effectiveness, attack rate and case fatality - the cost equivalence will be determined where the overall cost of vaccination equals that of treatment only.

*Breakeven cost of vaccination x Population = Overall cost _vaccination_ = Overall cost _treatment only_*

For the stockpiling scenario, the number of stockpile cycles to the next pandemic was determined by the time to the pandemic and the vaccine shelf-life. Each stockpile cycle incurred full costs at the year of purchase, and this was discounted to the year 2007. In addition, the net costs and benefits were calculated during the assumed year when the pandemic occurred and discounted to 2007 dollars.

**Results and discussion**

The one-way sensitivity analysis is shown in Figure S1 (the top 16 variables of impact are shown, the rest of the variables were analyzed but are less consequential and are not represented). The variable with the greatest impact on the outcome of overall cost-benefit was the pandemic’s case fatality followed by the attack rate. This showed that the key pandemic parameters are the most important when considering the strategy to adopt, and policy makers have to consider the perceived risks and impact of the next pandemic when deciding on the investment on preparedness. The variables of outpatient days lost and value of a day lost was also substantial, and these are related to the severity of the pandemic since the outpatient days lost is related to the clinical attack rate. A pandemic with high attach rate and morbidity will result in substantial costs even with low case-fatality; however, a pandemic with high case-fatality will result in the most costs.

The key vaccine parameters of cost, efficacy, and strain mismatch also had a substantial impact on the outcome. Policy makers will therefore have to consider the robustness of vaccine technologies available before embarking on such costly programs. The other key parameters of significance were the oseltamivir treatment parameters, which are equivalent in both treatment only and vaccination strategies and therefore not directly relevant to this comparison.

Surprisingly, healthcare costs featured low on the list of parameters that resulted in substantial impact to the outcome. This is because the healthcare costs are either one time costs (for outpatient treatment) or are only relevant for a small proportion of hospitalized patients, compared to days lost from work which applied across all infected individuals.

As shown in Table S1, for the entire population, even for vaccination within the fist stockpile where additional stockpile cycle costs are not incurred, vaccine efficacy and strain matching had a substantial impact on the outcomes. At high vaccine mismatch levels of more than 50%, vaccination is not a cost-beneficial strategy even if the vaccine if 100% efficacious against the matched strains. Similarly, the cost-effectiveness ratio is below the mean adult earnings of about USD$0.86m in many scenarios. This scenario of immediate vaccination is only cost-beneficial at high vaccine efficacy levels of more than 50% and low strain mismatch of less than 40%.

Once strategies of stockpiling are included (Table S2a and S2b), the mean cost-benefit becomes less compelling. Even the least costly scenario of 5 year shelf-life and time to a pandemic of 10 years with 2 stockpile cycles, and 100% efficacy and strain match, result in net costs of more than USD$66.4m. Stockpiling on vaccines for the entire population for a mild 1957 or 1968 type pandemic is therefore not cost-beneficial. The most costly scenario of 3 year shelf-life and time to pandemic of 50 years is even more costly. This is due to the high cost of vaccines and the need to replace stockpiles because unlike anti-virals such as oseltamivir, vaccinations are less likely to be deployed post expiry.

Table S3 shows the annual insurance premium for strategies of time to pandemic of 0 (impending pandemic), 10, 30, and 50 years. The shelf-life and cost of vaccine are not included in the analysis because the insurance premium includes these parameters as part of the maximal sum to pay for the entire vaccination program. This means that if shelf-life of the vaccine is 3 years, the amount that policy makers would be willing to pay would be 3 times the annual insurance premium including costs to stockpile and administer the vaccine and possible side-effects. Using the overall effectiveness of the vaccine as the variable parameter, the annual insurance premium increases with increasing vaccine effectiveness. The probability to the next pandemic of 1/10, 1/30, and 1/50 years reduces the annual insurance premium to be considered. At effectiveness levels of more than 50%, the insurance premium is about USD$46.4 or more, which matches the current estimated vaccination costs. If only one dose was needed and costs are reduced to seasonal vaccination costs of about USD$6.6, immediate vaccination is almost always cost-effective. Even under the lower cost of USD$6.6, stockpiling strategies were cost-beneficial only with high vaccine effectiveness of >60%. If the probability of the pandemic occurring only over 50 years is considered, stockpiling may only effective under simultaneous conditions of low vaccine cost and high vaccine effectiveness.

Table S4 examines the changes to the annual insurance premium with changes in the pandemic’s attack rate. If the cost of vaccination was similar to seasonal influenza vaccines at USD$6.6, vaccination would be cost-effective across almost all vaccine effectiveness and pandemic attack rates. Even under the lower cost of USD$6.6, stockpiling strategies were cost-beneficial only with high vaccine effectiveness of >60% at any attack rate, or if high attack rates were >40%. If the probability of the pandemic occurring only over 50 years is considered, stockpiling may only effective under simultaneous conditions of low vaccine cost, high vaccine effectiveness, and high attack rates.

Table S5 shows the mean annual insurance across a range of case-fatality rates. At 1957 and 1968 pandemic case-fatality rates of between 0.4 to 0.6%, the annual insurance rates are low, especially for the annual probability of a pandemic of 1/30 and 1/50, where it matches the seasonal influenza costs of USD$6.6 only if the vaccine effectiveness is more than 60-80%. At high case-fatality rates of 5% which is the upper estimate of the 1918 pandemic in certain regions, stockpiling of vaccines becomes a reasonable strategy across almost all stockpiling scenarios and vaccine effectiveness levels. From the seasonal influenza case-fatality of 0.1% to the high case-fatality of 5%, the annual insurance premium increased ten fold.

Table S6 compares the annual insurance premium based on the separate age and risk groups in a pandemic similar to the 1957 and 1968 pandemic. This is an important comparison because vaccination for the entire population may not be cost-effective in certain conditions as shown above. This sub-population analysis considered the annual insurance premium when only the sub-population was vaccinated. As evident, protection of any of the high-risk sub-populations commanded a higher insurance premium compared to any of the low-risk sub-populations. For the high-risk sub-population, children commanded the highest premiums followed by adults and the elderly. For high-risk children, most of the scenarios provided sufficient insurance premiums to justify stockpiling vaccines that have a shelf-life of 3 to 5 years at seasonal vaccine costs of USD$6.6. However, targeting the elderly resulted in the lowest cost per life saved, as well as the most lives saved by vaccination compared to treatment-only. This is because there were more deaths among the elderly, even though they had less direct economic value.

For the low-risk sub-population, the elderly commanded the highest premiums followed by adults and children (Table S7). Stockpiling of vaccines for these low-risk sub-populations commanded low insurance premiums, and was cost-effective at USD$6.6 only if the vaccine effectiveness was high.

Long-term stockpiling on vaccines is expensive and may not be cost-effective at the population level for mild pandemics. However, stockpiling is cost-beneficial if insurance against a severe pandemic is the main priority, if specific high-risk groups are targeted, and if cheap and effective vaccines are available through research and development.

References

1. Lee VJ, Phua KH, Chen MI, Chow A, Ma S, Goh KT, and Leo YS (2006) Economics of neuraminidase inhibitor stockpiling for pandemic influenza, Singapore. Emerg Infect Dis. 12: 95-102.
2. Balicer RD, Huerta M, Davidovitch N, Grotto I (2005) Cost-benefit of stockpiling drugs for influenza pandemic. Emerg Infect Dis. 11: 1280–1282.
3. Meltzer MI, Cox NJ, Fukuda K (1999) The Economic Impact of Pandemic Influenza in the United States: Priorities for Intervention. Emerg Infect Dis. 5: 659-671.

**Figure Legends**

**Figure S1 – Sensitivity of outcome (overall cost-benefit) to changes in input variables***

* “Case fatality” is the case fatality rate from influenza. “AR” is the attack rate of influenza during a pandemic. “Vacc cost” is the cost of vaccination. “Out Pt Lost” is the days lost from outpatient influenza. “Strain mis-match” is the mismatch of the vaccine strain to the pandemic strain, rendering it ineffective. “Eff. Of vacc” is the effectiveness of vaccine. “Sought Vac” is the proportion of people who sought vaccination early. “VO Lost day” is the value of a lost work day. “Rx Mort Red” is the reduction in mortality on those who sought early treatment. “Sought Rx” is the proportion who sought early treatment. “Rx Day Gain” is the lost days gained on early treatment. “Dr Cost” is the cost physician consultation cost. “Hosp rate” is the hospitalization rate from influenza. “LOS” is the mean length of stay in hospital. “Hosp Cost” is the mean cost of hospitalization. “Rx Hosp Red” is the reduction in hospitalization rate on those who sought early treatment.

**Table S1 – Costs and outcomes for vaccination within first stockpile with changes in vaccine efficacy and strain mismatch *†**

| **Cost benefit (millions USD$)** | | | | | |
| --- | --- | --- | --- | --- | --- |
| **Strain mismatch** | | | | | |
| **Vaccine Efficacy** | **0.0** | **0.2** | **0.4** | **0.6** | **0.8** |
| 0.2 | 103 (66, 128) | 116 (87, 138) | 126 (105, 142) | 139 (124, 150) | 151 (144, 157) |
| 0.4 | 39 (-35, 90) | 65 (9, 109) | 86 (44, 118) | 111 (81, 134) | 135 (121, 147) |
| 0.6 | -24 (-136, 52) | 14 (-70, 80) | 46 (-17, 93) | 83 (38, 118) | 119 (98, 137) |
| 0.8 | -88 (-237, 14) | -37 (-149, 51) | 5 (-78, 69) | 56 (-4, 102) | 104 (75, 127) |
| 1.0 | -152 (-338, -24) | -88 (-228, 22) | -35 (-139, 45) | 28 (-47, 86) | 88 (52, 117) |
|  |  |  |  |  |  |
| **Lives saved** | | | | | |
| **Strain mismatch** | | | | | |
| **Vaccine Efficacy** | **0.0** | **0.2** | **0.4** | **0.6** | **0.8** |
| 0.2 | 82 (41, 138) | 64 (32, 108) | 50 (25, 85) | 33 (17, 54) | 16 (8, 26) |
| 0.4 | 165 (82, 277) | 129 (63,215 0 | 101 (51, 171) | 65 (33, 109) | 32 (17, 53) |
| 0.6 | 247 (123, 415) | 193 (95, 323) | 151 (76,256) | 98 (50, 163) | 48 (25, 79) |
| 0.8 | 330 (165, 553) | 258 (127,431) | 201 (102, 341) | 130 (66, 218) | 65 (33,105) |
| 1.0 | 412 (206, 692) | 322 (158, 539) | 252 (127, 426) | 163 (83, 272) | 81 (42, 132) |
|  |  |  |  |  |  |
| **Cost per life saved (millions, USD$)** | | | | | |
| **Strain mismatch** | | | | | |
| **Vaccine Efficacy** | **0.0** | **0.2** | **0.4** | **0.6** | **0.8** |
| 0.2 | 1.78 (0.86, 3.19) | 2.42 (1.16, 4.37) | 3.24 (1.62, 5.81) | 5.23 (2.72, 9.05) | 10.94 (5.85, 18.72) |
| 0.4 | 0.63 (0.17, 1.23) | 0.94 (0.37, 1.81) | 1.34 (0.60, 2.45) | 2.31 (1.15, 4.09) | 5.10 (2.69, 8.80) |
| 0.6 | 0.24 (-0.12, 0.64) | 0.44 (0.04, 1.00) | 0.71 (0.23, 1.41) | 1.34 (0.60, 2.50) | 3.15 (1.60, 5.52) |
| 0.8 | 0.05 (-0.29, 0.36) | 0.2 (-0.18, 0.61) | 0.39 (0.01, 0.88) | 0.85 (0.32, 1.71) | 2.18 (1.08, 3.87) |
| 1.0 | -0.06 (-0.41, 0.21) | 0.05 (-0.32, 0.39) | 0.20 (-0.14, 0.58) | 0.56 (0.12, 1.22) | 1.59 (0.75, 2.85) |

*Mean values are shown with 5^th^ and 95^th^ percentiles.

†All healthcare costs are in 2007 USD dollars (2007 exchange rate, USD$1: SGD$1.507).

**Table S2a – Costs and outcomes for stockpiling of vaccines, 10 years to pandemic and shelf-life of 5 years*†**

| **Cost benefit (millions USD$)** | | | | | | |
| --- | --- | --- | --- | --- | --- | --- |
| **Strain mismatch** | | | | | | |
| **Vac. Eff.** | **0.0** | **0.2** | **0,4** | **0.6** | **0.8** | **1.0** |
| 0.0 | 310 (310, 310) | 310 (310, 310) | 310 (310, 310) | 310 (310, 310) | 310 (310, 310) | 310 (310, 310) |
| 0.2 | 263 (235, 283) | 271 (251, 288) | 281 (265, 293) | 289 (278, 298) | 298 (292, 303) | 310 (310, 310) |
| 0.4 | 215 (160, 256) | 233 (192, 265) | 251 (220, 275) | 269 (247, 285) | 286 (275, 295) | 310 (310, 310) |
| 0.6 | 168 (85, 230) | 194 (133, 243) | 222 (175, 258) | 248 (215, 273) | 274 (257, 288) | 310 (310, 310) |
| 0.8 | 121 (10, 203) | 156 (74, 221) | 192 (130, 240) | 227 (183, 261) | 262 (239, 281) | 310 (310, 310) |
| 1.0 | 71 (-51, 172) | 117 (-1, 203) | 163 (86, 223) | 206 (152, 249) | 250 (222, 274) | 310 (310, 310) |
|  |  |  |  |  |  |  |
| **Lives saved** | | | | | | |
| **Strain mismatch** | | | | | | |
| **Vac. Eff.** | **0.0** | **0.2** | **0,4** | **0.6** | **0.8** | **1.0** |
| 0.0 | - | - | - | - | - | - |
| 0.2 | 82 (42, 133) | 65 (33, 112) | 49 (24, 80) | 32 (17, 53) | 16 (8, 27) | - |
| 0.4 | 164 (83, 266) | 130 (65, 223) | 99 (49, 160) | 65 (33, 106) | 33 (16, 54) | - |
| 0.6 | 245 (125, 399) | 194 (98, 335) | 148 (73, 239) | 97 (50, 160) | 49 (24, 82) | - |
| 0.8 | 327 (167, 532) | 259 (131, 446) | 197 (98, 319) | 129 (66, 213) | 66 (32, 109) | - |
| 1.0 | 413 (204, 679) | 329 (171, 552) | 89 (122, 399) | 161 (83, 266) | 82 (41, 136) | - |
|  |  |  |  |  |  |  |
| **Cost per life saved (millions, USD$)** | | | | | | |
| **Strain mismatch** | | | | | | |
| **Vac. Eff.** | **0.0** | **0.2** | **0,4** | **0.6** | **0.8** | **1.0** |
| 0.0 | **-** | **-** | **-** | **-** | **-** | **-** |
| 0.2 | 3.92 (2.04, 6.89) | 5.01 (2.53, 8.75) | 6.75 (3.65, 11.8) | 10.4 (5.53, 18.1) | 20.95 (11.0, 37.4) | **-** |
| 0.4 | 1.76 (0.89, 3.17) | 2.31 (1.14, 4.10) | 3.16 (1.67, 5.50) | 4.98 (2.65, 8.72) | 10.2 (5.34, 18.16) | **-** |
| 0.6 | 1.04 (0.49, 1.94) | 1.41 (0.68, 2.51) | 1.97 (1.02, 3.48) | 3.17 (1.68, 5.57) | 6.61 (3.44, 11.73) | **-** |
| 0.8 | 0.68 (0.27, 1.33) | 0.95 (0.43, 1.76) | 1.37 (0.68, 2.45) | 2.26 (1.19, 4.04) | 4.82 (2.5, 8.61) | **-** |
| 1.0 | 0.46 (0.14, 0.92) | 0.67 (0.26, 1.28) | 1.02 (0.48, 1.87) | 1.72 (0.89, 3.05) | 3.74 (1.91, 6.72) | **-** |

*Mean values are shown with 5^th^ and 95^th^ percentiles.

†All healthcare costs are in 2007 USD dollars (2007 exchange rate, USD$1: SGD$1.507).

**Table S2b – Costs and outcomes for stockpiling of vaccines, 50 years to pandemic and shelf-life of 3 years*†**

| **Cost benefit (millions, USD$)** | | | | | | |
| --- | --- | --- | --- | --- | --- | --- |
| **Strain mismatch** | | | | | | |
| **Vac. Eff.** | **0.0** | **0.2** | **0,4** | **0.6** | **0.8** | **1.0** |
| 0.0 | 1527 (1527, 1527) | 1527 (1527, 1527) | 1527 (1527, 1527) | 1527 (1527, 1527) | 1527 (1527, 1527) | 1527 (1527, 1527) |
| 0.2 | 1512 (1505, 1518) | 1515 (1508, 1520) | 1518 (1513, 1522) | 1521 (1517, 1514) | 1523 (1522, 1525) | 1527 (1527, 1527) |
| 0.4 | 1498 (1483, 1510) | 1503 (1490, 1513) | 1509 (1499, 1516) | 1514 (1508, 1519) | 1520 (1516, 1522) | 1527 (1527, 1527) |
| 0.6 | 1483 (1461, 1501) | 1491 (1471, 1506) | 1500 (1486, 1511) | 1508 (1498, 1516) | 1516 (1511, 1520) | 1527 (1527, 1527) |
| 0.8 | 1469 (1439, 1492) | 1479 (1453, 1499) | 1491 (1472, 1505) | 1502 (1489, 1512) | 1512 (1505, 1518) | 1527 (1527, 1527) |
| 1.0 | 1454 (1417, 1483) | 1467 (1434, 1492) | 1482 (1458, 1500) | 1496 (1479, 1508) | 1509 (1500, 1516) | 1527 (1527, 1527) |
|  |  |  |  |  |  |  |
| **Lives saved** | | | | | | |
| **Strain mismatch** | | | | | | |
| **Vac. Eff.** | **0.0** | **0.2** | **0,4** | **0.6** | **0.8** | **1.0** |
| 0.0 | - | - | - | - | - | - |
| 0.2 | 82 (40, 139) | 67 (34, 113) | 50 (25, 84) | 33 (17, 55) | 17 (9, 28) | - |
| 0.4 | 164 (80, 278) | 134 (68, 227) | 99 (51, 167) | 66 (34, 109) | 33 (17,56) | - |
| 0.6 | 246 (120, 418) | 201 (102, 340) | 149 (76, 251) | 100 (52, 164) | 50 (26, 84) | - |
| 0.8 | 328 (160, 557) | 263 (136, 453) | 198 (102, 334) | 133 (69, 219) | 67 (35, 112) | - |
| 1.0 | 410 (200, 696) | 336 (170, 567) | 248 (127, 418) | 166 (86, 273) | 83 (43, 140) | - |
|  |  |  |  |  |  |  |
| **Cost per life saved (millions, USD$)** | | | | | | |
| **Strain mismatch** | | | | | | |
| **Vac. Eff.** | **0.0** | **0.2** | **0,4** | **0.6** | **0.8** | **1.0** |
| 0.0 | **-** | **-** | **-** | **-** | **-** | **-** |
| 0.2 | 21 (11, 38) | 26 (13, 44) | 35 (18, 59) | 52 (28, 88) | 104 (54, 175) | **-** |
| 0.4 | 11 (5, 19) | 13 (7, 22) | 17 (9, 30) | 26 (14, 44) | 52 (27, 88) | **-** |
| 0.6 | 7 (4, 12) | 9 (4, 15) | 12 (6, 20) | 17 (9, 29) | 35 (18, 58) | **-** |
| 0.8 | 5 (3, 9) | 6 (3, 11) | 9 (4, 15) | 13 (7, 22) | 26 (13,44) | **-** |
| 1.0 | 4 (2, 7) | 5 (3,9) | 7 (4, 12) | 10 (5, 17) | 21 (11, 35) | **-** |

*Mean values are shown with 5^th^ and 95^th^ percentiles.

†All healthcare costs are in 2007 USD dollars (2007 exchange rate, USD$1: SGD$1.507).

**Table S3 – Annual insurance premium for pandemic scenarios with changes in vaccine effectiveness *†**

| **Annual insurance (USD$)** | | | | |
| --- | --- | --- | --- | --- |
| **Eff. Of vaccine** | **Impending pandemic** | **10 years** | **30 years** | **50 years** |
| 0.1 | 8.88 (5.19, 13.9) | 0.89 (0.52, 1.39) | 0.3 (0.17, 0.46) | 0.18 (0.10, 0.28) |
| 0.2 | 17.74 (10.36, 27.79) | 1.77 (1.04, 2.78) | 0.59 (0.35, 0.93) | 0.35 (0.21, 0.56) |
| 0.3 | 26.6 (15.54, 41.69) | 2.66 (1.55, 4.17) | 0.89 (0.52, 1.39) | 0.53 (0.3, 0.83) |
| 0.4 | 35.46 (20.71, 55.58) | 3.55 (2.07, 5.56) | 1.18 (0.69, 1.85) | 0.71 (0.41, 1.11) |
| 0.5 | 44.32 (25.89, 69.47) | 4.43 (2.59, 6.95) | 1.48 (0.86, 2.32) | 0.89 (0.52, 1.39) |
| 0.6 | 53.18 (31.06, 83.36) | 5.32 (3.11, 8.34) | 1.77 (1.04, 2.78) | 1.06 (0.62, 1.67) |
| 0.7 | 62.04 (36.24, 97.24) | 6.20 (3.62, 9.72) | 2.07 (1.21, 3.24) | 1.24 (0.72, 1.94) |
| 0.8 | 70.9 (41.41, 111.13) | 7.09 (4.14, 11.11) | 2.36 (1.38, 3.70) | 1.42 (0.83, 2.22) |
| 0.9 | 79.76 (46.59, 125.02) | 7.98 (4.66, 12.5) | 2.66 (1.55, 4.17) | 1.60 (0.93, 2.50) |

*Mean values are shown with 5^th^ and 95^th^ percentiles.

†All healthcare costs are in 2007 USD dollars (2007 exchange rate, USD$1: SGD$1.507).

**Table S4 – Annual insurance premium for pandemic scenarios with changes in vaccine effectiveness and attack rate*†**

| **Impending pandemic** | | | | | |
| --- | --- | --- | --- | --- | --- |
| **Vaccine effectiveness** | | | | | |
| **Attack rate** | **0.2** |  | **0.4** | **0.6** | **0.8** |
| 0.1 | 6.02 (4.13, 8.33) |  | 11.8 (8.03, 16.2) | 17.9 (12.5, 24.3) | 23.8 (16.4, 32.6) |
| 0.3 | 18.1 (12.4, 25.0) |  | 35.5 ( 24.1, 48.5) | 53.8 (37.5, 72.7) | 71.4 (49.3, 97.9) |
| 0.5 | 30.1 (20.7, 41.7) |  | 59.2 (40.2, 80.9) | 89.6 (62.5, 121.2) | 118.9 (82.2, 163.2) |
|  |  |  |  |  |  |
| **Probability of pandemic occurring spread over 10 years** | | | | | |
| **Vaccine effectiveness** | | | | | |
| **Attack rate** | **0.2** |  | **0.4** | **0.6** | **0.8** |
| 0.1 | 0.60 (0.41, 0.83) |  | 1.18 (0.80, 1.62) | 1.79 (1.25, 2.42) | 2.38 (1.64, 3.26) |
| 0.3 | 1.81 (1.24, 2.50) |  | 3.55 (2.41, 4.85) | 5.37 (3.75, 7.27) | 7.14 (4.93, 9.79) |
| 0.5 | 3.01 (2.07, 4.16) |  | 5.92 (4.02, 8.09) | 8.96 (6.25, 12.1) | 11.9 (8.22, 16.3) |
|  |  |  |  |  |  |
| **Probability of pandemic occurring spread over 30 years** | | | | | |
| **Vaccine effectiveness** | | | | | |
| **Attack rate** | **0.2** |  | **0.4** | **0.6** | **0.8** |
| 0.1 | 0.20 (0.14, 0.28) |  | 0.39 (0.27, 0.54) | 0.60 (0.42, 0.81) | 0.79 (0.55, 1.09) |
| 0.3 | 0.60 (0.41, 0.83) |  | 1.18 (0.80, 1.62) | 1.79 (1.25, 2.42) | 2.38 (1.64, 3.26) |
| 0.5 | 1.00 (0.69, 1.39) |  | 1.97 (1.34, 2.70) | 2.99 (2.08, 4.04) | 3.96 (2.74, 5.44) |
|  |  |  |  |  |  |
| **Probability of pandemic occurring spread over 50 years** | | | | | |
| **Vaccine effectiveness** | | | | | |
| **Attack rate** | **0.2** |  | **0.4** | **0.6** | **0.8** |
| 0.1 | 0.123 (0.08, 0.17) |  | 0.24 (0.16, 0.32) | 0.36 (0.25, 0.48) | 0.48 (0.33, 0.65) |
| 0.3 | 0.36 (0.25, 0.50) |  | 0.71 (0.48, 0.97) | 1.07 (0.75, 1.45) | 1.43 (0.99, 1.95) |
| 0.5 | 0.60 (0.41, 0.83) |  | 1.18 (0.80, 1.62) | 1.79 (1.25, 2.42) | 2.38 (1.64, 3.26) |

*Mean values are shown with 5^th^ and 95^th^ percentiles.

†All healthcare costs are in 2007 USD dollars (2007 exchange rate, USD$1: SGD$1.507).

**Table S5 – Annual insurance premium for pandemic scenarios with changes in vaccine effectiveness and mortality rate*†**

| **Annual insurance (USD$)** | | | | | | | | | | | | | | | | |
| --- | --- | --- | --- | --- | --- | --- | --- | --- | --- | --- | --- | --- | --- | --- | --- | --- |
|  | **Impending pandemic** | | |  | **10 years** | |  |  | **30 years** | |  |  | **50 years** | |  |  |
| **Effectiveness of vaccine** | | | | | | | | | | | | | | | | |
| **Mortality rate** | **0.2** | **0.4** | **0.6** | **0.8** | **0.2** | **0.4** | **0.6** | **0.8** | **0.2** | **0.4** | **0.6** | **0.8** | **0.2** | **0.4** | **0.6** | **0.8** |
| 0.00% | 10.71 | 21.26 | 32.45 | 42.48 | 1.07 | 2.13 | 3.25 | 4.25 | 0.36 | 0.71 | 1.08 | 1.42 | 0.21 | 0.43 | 0.65 | 0.85 |
| 0.20% | 15.40 | 30.61 | 46.50 | 60.96 | 1.54 | 3.06 | 4.65 | 6.10 | 0.51 | 1.02 | 1.55 | 2.03 | 0.31 | 0.61 | 0.93 | 1.22 |
| 0.40% | 20.08 | 39.96 | 60.54 | 79.44 | 2.01 | 4.00 | 6.05 | 7.94 | 0.67 | 1.33 | 2.02 | 2.65 | 0.40 | 0.80 | 1.21 | 1.59 |
| 0.60% | 24.77 | 49.31 | 74.58 | 97.92 | 2.48 | 4.93 | 7.46 | 9.79 | 0.83 | 1.64 | 2.49 | 3.26 | 0.50 | 0.99 | 1.49 | 1.96 |
| 0.80% | 29.45 | 58.66 | 88.63 | 116.40 | 2.95 | 5.87 | 8.86 | 11.64 | 0.98 | 1.96 | 2.95 | 3.88 | 0.59 | 1.17 | 1.77 | 2.33 |
| 1.00% | 34.14 | 68.01 | 102.67 | 134.88 | 3.41 | 6.80 | 10.27 | 13.49 | 1.14 | 2.27 | 3.42 | 4.50 | 0.68 | 1.36 | 2.05 | 2.70 |
| 1.20% | 38.82 | 77.35 | 116.71 | 153.36 | 3.88 | 7.74 | 11.67 | 15.34 | 1.29 | 2.58 | 3.89 | 5.11 | 0.78 | 1.55 | 2.33 | 3.07 |
| 1.40% | 43.51 | 86.70 | 130.75 | 171.84 | 4.35 | 8.67 | 13.08 | 17.18 | 1.45 | 2.89 | 4.36 | 5.73 | 0.87 | 1.73 | 2.62 | 3.44 |
| 1.60% | 48.19 | 96.05 | 144.80 | 190.32 | 4.82 | 9.61 | 14.48 | 19.03 | 1.61 | 3.20 | 4.83 | 6.34 | 0.96 | 1.92 | 2.90 | 3.81 |
| 1.80% | 52.88 | 105.40 | 158.84 | 208.80 | 5.29 | 10.54 | 15.88 | 20.88 | 1.76 | 3.51 | 5.29 | 6.96 | 1.06 | 2.11 | 3.18 | 4.18 |
| 2.00% | 57.57 | 114.73 | 172.14 | 227.42 | 5.76 | 11.47 | 17.21 | 22.74 | 1.92 | 3.82 | 5.74 | 7.58 | 1.15 | 2.29 | 3.44 | 4.55 |
| 2.20% | 62.25 | 124.06 | 186.09 | 245.93 | 6.22 | 12.41 | 18.61 | 24.59 | 2.07 | 4.14 | 6.20 | 8.20 | 1.24 | 2.48 | 3.72 | 4.92 |
| 2.40% | 66.93 | 133.39 | 200.05 | 264.44 | 6.69 | 13.34 | 20.00 | 26.44 | 2.23 | 4.45 | 6.67 | 8.81 | 1.34 | 2.67 | 4.00 | 5.29 |
| 2.60% | 71.61 | 142.72 | 214.00 | 282.94 | 7.16 | 14.27 | 21.40 | 28.29 | 2.39 | 4.76 | 7.13 | 9.43 | 1.43 | 2.85 | 4.28 | 5.66 |
| 2.80% | 76.28 | 152.05 | 227.95 | 301.45 | 7.63 | 15.20 | 22.80 | 30.14 | 2.54 | 5.07 | 7.60 | 10.05 | 1.53 | 3.04 | 4.56 | 6.03 |
| 3.00% | 80.96 | 161.37 | 241.91 | 319.96 | 8.10 | 16.14 | 24.19 | 32.00 | 2.70 | 5.38 | 8.06 | 10.67 | 1.62 | 3.23 | 4.84 | 6.40 |
| 3.20% | 85.64 | 170.70 | 255.86 | 338.46 | 8.56 | 17.07 | 25.59 | 33.85 | 2.85 | 5.69 | 8.53 | 11.28 | 1.71 | 3.41 | 5.12 | 6.77 |
| 3.40% | 90.32 | 180.03 | 269.82 | 356.97 | 9.03 | 18.00 | 26.98 | 35.70 | 3.01 | 6.00 | 8.99 | 11.90 | 1.81 | 3.60 | 5.40 | 7.14 |
| 3.60% | 95.00 | 189.36 | 283.77 | 375.47 | 9.50 | 18.94 | 28.38 | 37.55 | 3.17 | 6.31 | 9.46 | 12.52 | 1.90 | 3.79 | 5.68 | 7.51 |
| 3.80% | 99.68 | 198.69 | 297.72 | 393.98 | 9.97 | 19.87 | 29.77 | 39.40 | 3.32 | 6.62 | 9.92 | 13.13 | 1.99 | 3.97 | 5.95 | 7.88 |
| 4.00% | 102.74 | 205.58 | 312.22 | 416.18 | 10.27 | 20.56 | 31.22 | 41.62 | 3.42 | 6.85 | 10.41 | 13.87 | 2.05 | 4.11 | 6.24 | 8.32 |
| 4.20% | 107.35 | 214.80 | 326.23 | 434.82 | 10.73 | 21.48 | 32.62 | 43.48 | 3.58 | 7.16 | 10.87 | 14.49 | 2.15 | 4.30 | 6.52 | 8.70 |
| 4.40% | 111.95 | 224.03 | 340.23 | 453.47 | 11.20 | 22.40 | 34.02 | 45.35 | 3.73 | 7.47 | 11.34 | 15.12 | 2.24 | 4.48 | 6.80 | 9.07 |
| 4.60% | 116.56 | 233.25 | 354.24 | 472.12 | 11.66 | 23.33 | 35.42 | 47.21 | 3.89 | 7.78 | 11.81 | 15.74 | 2.33 | 4.67 | 7.08 | 9.44 |
| 4.80% | 121.17 | 242.47 | 368.25 | 490.76 | 12.12 | 24.25 | 36.82 | 49.08 | 4.04 | 8.08 | 12.27 | 16.36 | 2.42 | 4.85 | 7.36 | 9.82 |
| 5.00% | 125.78 | 251.70 | 382.25 | 509.41 | 12.58 | 25.17 | 38.23 | 50.94 | 4.19 | 8.39 | 12.74 | 16.98 | 2.52 | 5.03 | 7.65 | 10.19 |

*Mean values are shown with 5^th^ and 95^th^ percentiles.

†All healthcare costs are in 2007 USD dollars (2007 exchange rate, USD$1: SGD$1.507).

**Table S6 – Annual insurance premium for pandemic scenarios with changes in vaccine effectiveness, by age and risk groups*†**

| **Impending pandemic** | | | | | | | | | | | | |
| --- | --- | --- | --- | --- | --- | --- | --- | --- | --- | --- | --- | --- |
| **Vaccine effectiveness** | **0.2** |  |  | **0.4** |  |  | **0.6** |  |  | **0.8** |  |  |
| **Risk Groups** | **Mean** | **5%** | **95%** | **Mean** | **5%** | **95%** | **Mean** | **5%** | **95%** | **Mean** | **5%** | **95%** |
| Low risk - children | 9.43 | 7.18 | 5.30 | 18.87 | 14.36 | 10.59 | 28.30 | 21.53 | 15.89 | 37.73 | 28.71 | 21.19 |
| Low risk - adult | 12.79 | 10.44 | 7.10 | 25.59 | 20.88 | 14.19 | 38.38 | 31.32 | 21.29 | 51.18 | 41.76 | 28.39 |
| Low risk - elderly | 14.80 | 11.86 | 8.75 | 29.60 | 23.72 | 17.49 | 44.39 | 35.58 | 26.24 | 59.19 | 47.44 | 34.99 |
| High risk - children | 67.37 | 89.33 | 47.47 | 134.73 | 178.66 | 94.93 | 202.10 | 267.98 | 142.40 | 269.47 | 357.31 | 189.86 |
| High risk - adult | 57.32 | 58.89 | 37.96 | 114.63 | 117.78 | 75.93 | 171.95 | 176.67 | 113.89 | 229.26 | 235.56 | 151.86 |
| High risk - elderly | 45.46 | 45.34 | 29.02 | 90.91 | 90.68 | 58.03 | 136.37 | 136.02 | 87.05 | 181.82 | 181.36 | 116.07 |
| **Probability of pandemic occurring spread over 10 years** | | | | | | | | | | | | |
| **Vaccine effectiveness** | **0.2** |  |  | **0.4** |  |  | **0.6** |  |  | **0.8** |  |  |
| **Risk Groups** | **Mean** | **5%** | **95%** | **Mean** | **5%** | **95%** | **Mean** | **5%** | **95%** | **Mean** | **5%** | **95%** |
| Low risk - children | 0.94 | 0.72 | 0.53 | 1.89 | 1.44 | 1.06 | 2.83 | 2.15 | 1.59 | 3.77 | 2.87 | 2.12 |
| Low risk - adult | 1.28 | 1.04 | 0.71 | 2.56 | 2.09 | 1.42 | 3.84 | 3.13 | 2.13 | 5.12 | 4.18 | 2.84 |
| Low risk - elderly | 1.48 | 1.19 | 0.87 | 2.96 | 2.37 | 1.75 | 4.44 | 3.56 | 2.62 | 5.92 | 4.74 | 3.50 |
| High risk - children | 6.74 | 8.93 | 4.75 | 13.47 | 17.87 | 9.49 | 20.21 | 26.80 | 14.24 | 26.95 | 35.73 | 18.99 |
| High risk - adult | 5.73 | 5.89 | 3.80 | 11.46 | 11.78 | 7.59 | 17.19 | 17.67 | 11.39 | 22.93 | 23.56 | 15.19 |
| High risk - elderly | 4.55 | 4.53 | 2.90 | 9.09 | 9.07 | 5.80 | 13.64 | 13.60 | 8.71 | 18.18 | 18.14 | 11.61 |
| **Probability of pandemic occurring spread over 30 years** | | | | | | | | | | | | |
| **Vaccine effectiveness** | **0.2** | | | **0.4** | | | **0.6** | | | **0.8** | | |
| **Risk Groups** | **Mean** | **5%** | **95%** | **Mean** | **5%** | **95%** | **Mean** | **5%** | **95%** | **Mean** | **5%** | **95%** |
| Low risk - children | 0.31 | 0.24 | 0.18 | 0.63 | 0.48 | 0.35 | 0.94 | 0.72 | 0.53 | 1.26 | 0.96 | 0.71 |
| Low risk - adult | 0.43 | 0.35 | 0.24 | 0.85 | 0.70 | 0.47 | 1.28 | 1.04 | 0.71 | 1.71 | 1.39 | 0.95 |
| Low risk - elderly | 0.49 | 0.40 | 0.29 | 0.99 | 0.79 | 0.58 | 1.48 | 1.19 | 0.87 | 1.97 | 1.58 | 1.17 |
| High risk - children | 2.25 | 2.98 | 1.58 | 4.49 | 5.96 | 3.16 | 6.74 | 8.93 | 4.75 | 8.98 | 11.91 | 6.33 |
| High risk - adult | 1.91 | 1.96 | 1.27 | 3.82 | 3.93 | 2.53 | 5.73 | 5.89 | 3.80 | 7.64 | 7.85 | 5.06 |
| High risk - elderly | 1.52 | 1.51 | 0.97 | 3.03 | 3.02 | 1.93 | 4.55 | 4.53 | 2.90 | 6.06 | 6.05 | 3.87 |
| **Probability of pandemic occurring spread over 50 years** | | | | | | | | | | | | |
| **Vaccine effectiveness** | **0.2** | | | **0.4** | | | **0.6** | | | **0.8** | | |
| **Risk Groups** | **Mean** | **5%** | **95%** | **Mean** | **5%** | **95%** | **Mean** | **5%** | **95%** | **Mean** | **5%** | **95%** |
| Low risk - children | 0.19 | 0.14 | 0.11 | 0.38 | 0.29 | 0.21 | 0.57 | 0.43 | 0.32 | 0.75 | 0.57 | 0.42 |
| Low risk - adult | 0.26 | 0.21 | 0.14 | 0.51 | 0.42 | 0.28 | 0.77 | 0.63 | 0.43 | 1.02 | 0.84 | 0.57 |
| Low risk - elderly | 0.30 | 0.24 | 0.17 | 0.59 | 0.47 | 0.35 | 0.89 | 0.71 | 0.52 | 1.18 | 0.95 | 0.70 |
| High risk - children | 1.35 | 1.79 | 0.95 | 2.69 | 3.57 | 1.90 | 4.04 | 5.36 | 2.85 | 5.39 | 7.15 | 3.80 |
| High risk - adult | 1.15 | 1.18 | 0.76 | 2.29 | 2.36 | 1.52 | 3.44 | 3.53 | 2.28 | 4.59 | 4.71 | 3.04 |
| High risk - elderly | 0.91 | 0.91 | 0.58 | 1.82 | 1.81 | 1.16 | 2.73 | 2.72 | 1.74 | 3.64 | 3.63 | 2.32 |

*Mean values are shown with 5^th^ and 95^th^ percentiles.

†All healthcare costs are in 2007 USD dollars (2007 exchange rate, USD$1: SGD$1.507).

**Table S7 – Costs and outcomes for vaccination within first stockpile with changes in vaccine effectiveness, by age and risk groups*†**

| **Cost benefit (millions USD$)** | | | | |
| --- | --- | --- | --- | --- |
| **Eff. Of vaccine** | | | | |
| **Risk Group** | **0.2** | **0.4** | **0.6** | **0.8** |
| Low risk - children | 23 (19, 26) | 17 (9, 23) | 11 (-1, 21) | 5 (-11, 18) |
| Low risk - adult | 79 (53, 95) | 49 (-2, 82) | 19 (-58, 69) | -11 (-113, 57) |
| Low risk - elderly | 6 (4, 8) | 3 (-1, 7) | 0 (-6, 5) | -2 (-11, 4) |
| High risk - children | -2 (-8, 2) | -6 (-19, 0) | -11 (-30, -1) | -16 (-40, -2) |
| High risk - adult | -3 (-18, 7) | -18 (-49, 2) | -34 (-80, -3) | -49 (-110, -8) |
| High risk - elderly | 0 (-4, 3) | -5 (-13, 1) | -10 (-23, -1) | -15 (-32, -3) |
|  |  |  |  |  |
| **Lives saved** | | | | |
| **Eff. Of vaccine** | | | | |
| **Risk Group** | **0.2** | **0.4** | **0.6** | **0.8** |
| Low risk - children | 1 (0, 2) | 2 (1, 3) | 2 (1, 5) | 3 (1, 6) |
| Low risk - adult | 3 (1, 5) | 5 (2, 10) | 8 (3, 15) | 10 (4, 20) |
| Low risk - elderly | 15 (4, 31) | 30 (9, 61) | 46 (13, 92) | 61 (17, 122) |
| High risk - children | 4 (1, 11) | 9 (2, 21) | 13 (3, 32) | 18 (4, 42) |
| High risk - adult | 14 (3, 30) | 28 (6, 61) | 42 (10, 91) | 56 (13, 122) |
| High risk - elderly | 44 (15, 86) | 89 (31, 171) | 133 (46, 257) | 178 (61, 343) |
|  |  |  |  |  |
| **Cost per life saved (millions, USD$)** | | | | |
| **Eff. Of vaccine** | | | | |
| **Risk Group** | **0.2** | **0.4** | **0.6** | **0.8** |
| Low risk - children | 38.65 (12.26, 86.95) | 15.19 (3.21, 37.01) | 7.37 (-0.28, 21.04) | 3.46 (-2.49, 13.34) |
| Low risk - adult | 41.24 (12.03, 93.65) | 14.12 (-0.29, 38.38) | 5.08 (-5.52, 21.01) | 0.55 (-8.83, 11.72) |
| Low risk - elderly | 0.66 (0.14, 1.81) | 0.23 (0.23, 0.77) | 0.09 (-0.06, 0.41) | 0.01 (-0.09, 0.23) |
| High risk - children | 0.08 (-0.71, 1.86) | -0.52 (-0.86, 0.24) | -0.72 (-0.92, -0.28) | -0.82 (-0.97, -0.51) |
| High risk - adult | 0.23 (-0.62, 2.22) | -0.49 (-0.86, 0.38) | -0.72 (-1, -0.22) | -0.84 (-1.1, -0.52) |
| High risk - elderly | 0.05 (-0.04, 0.21) | -0.03 (-0.07, 0.04) | -0.06 (-0.09, -0.01) | -0.07 (-0.09, -0.04) |

*Mean values are shown with 5^th^ and 95^th^ percentiles.

†All healthcare costs are in 2007 USD dollars (2007 exchange rate, USD$1: SGD$1.507).
