# Supplementary figures and images for: Economic Analysis of Pandemic Influenza Vaccination Strategies in Singapore
Source: PLoS One. 2009 Sep 22;4(9):e7108. doi: 10.1371/journal.pone.0007108 (PMC2743808; doi:10.1371/journal.pone.0007108)

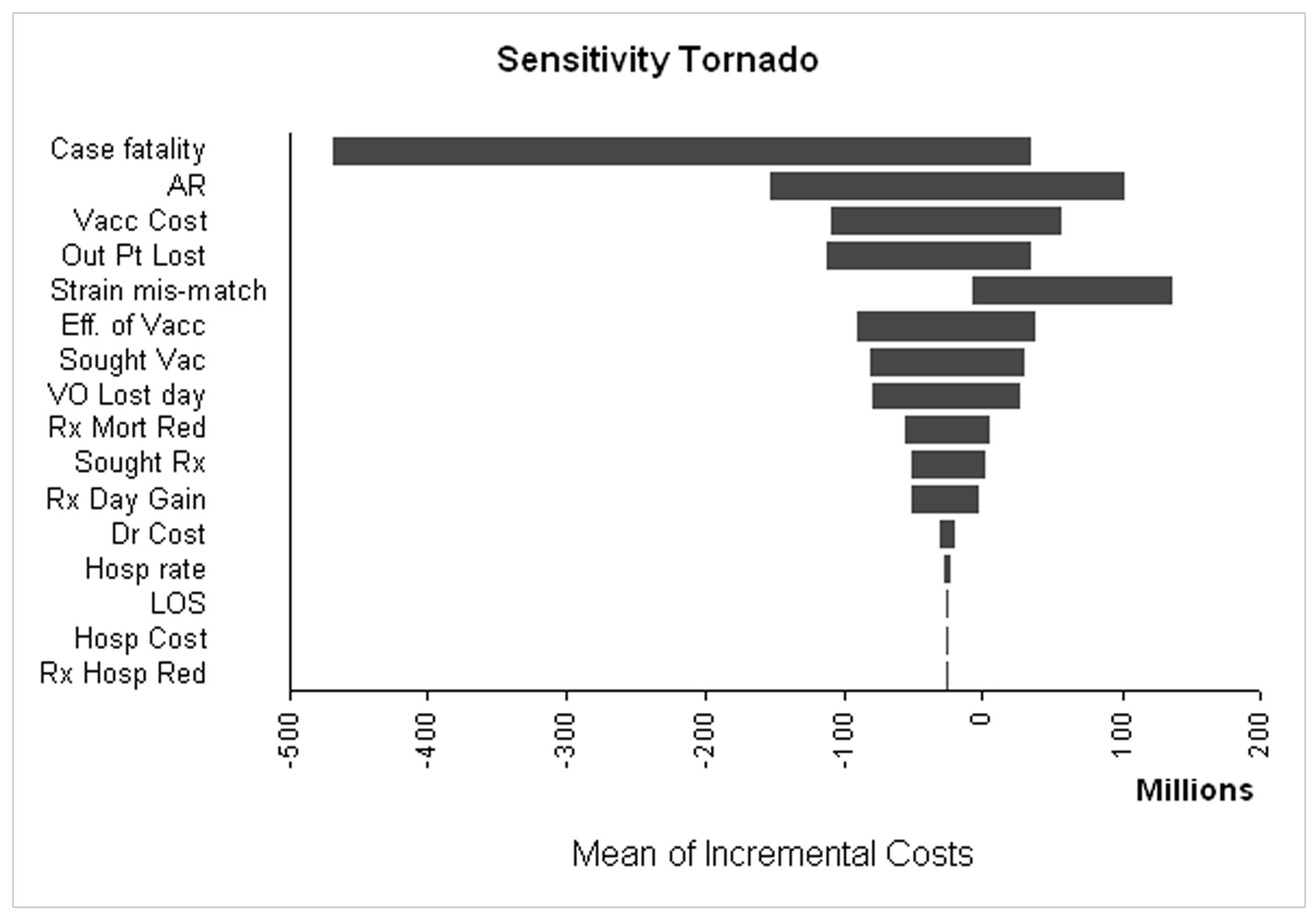

Supplement: Figure S1 — (0.43 MB TIF) [file pone.0007108.s002.tif]
